# Percepción del profesionalismo en personal médico latinoamericano en 2024: Una encuesta multinacional

**DOI:** 10.23938/ASSN.1112

**Published:** 2025-04-25

**Authors:** Raúl Emilio Real Delor, Alberto Guevara Tirado, Eduardo Enrique Chibas Muñoz, Ruth Stephany Benítez Penayo, Bárbara Girhfietl Caje Román, Jonatas de Oliveira Borba, Lucio Fabián Duarte Irala, Mirtha Sofía Fleitas Armoa, Elena Cecilia Franco Cáceres, Alberto Martins, Hugo Javier Peralta Echagüe, María Selva Vega Tande

**Affiliations:** 1 Universidad Privada del Este Facultad de Ciencias Médicas Asunción Paraguay; 2 Universidad Científica del Sur Facultad de Medicina Humana Lima Perú; 3 Universidad de Ciencias Médicas de Guantánamo Guantánamo Cuba

**Keywords:** Profesionalismo, Actitud del personal de salud, Mala conducta profesional, Personal de salud, Encuestas y cuestionarios, Professionalism, Attitude of Health Personnel, Professional misconduct, Health Personnel, Surveys and questionnaires

## Abstract

**Fundamento:**

El profesionalismo médico es el conjunto de valores, actitudes y comportamientos orientados al servicio del paciente y de la sociedad. El objetivo es evaluar la percepción del profesionalismo en personal médico latinoamericano en 2024.

**Metodología:**

Estudio observacional, transversal, en personal médico de diferentes especialidades latinoamericano. Se realizó una encuesta telemática que incluía variables sociodemográficas (edad, sexo, país, especialidad) y el cuestionario de profesionalismo de Kwon y colaboradores modificado (siete dimensiones y 43 preguntas).

**Resultados:**

Respondieron 424 personas, con mediana de edad 40 años, el 57,6% mujeres y 69,8% de especialidad clínica, procedentes de Paraguay (72,9%), Perú (24,3%) y Cuba (2,8%). La puntuación 1,223 sobre 4 discriminó el 40% de puntuaciones con menor profesionalismo. Los indicadores con menor puntuación (menos frecuentes) fueron *Recomiendo drogas ilegales con fines recreativos* y *Mantengo contacto corporal inapropiado durante el examen físico*. Los de mayor puntación fueron *Llego tarde al trabajo* y *Paso por alto los errores médicos de los colegas*. Los hombres mostraron significativamente menos profesionalismo respecto a tres indicadores: *Mantengo contacto corporal inapropiado durante el examen físico*, *Trato a otros profesionales de la salud de manera abusiva*, y *Ejerzo algún tipo de discriminación con colegas y pacientes* (efecto ajustado).

**Conclusiones:**

Se han detectado 16 indicadores con puntuaciones significativamente relacionadas con el nivel de profesionalismo auto percibido por los médicos, cuatro de ellos difieren significativamente por sexo. Recomendamos seguir aplicando este tipo de encuestas a otros niveles y aplicar estrategias a nivel universitario para generar conciencia sobre los valores del profesionalismo.

## Abstract (en)

**Background:**

Medical professionalism encompasses the values, attitudes, and behaviors that prioritize the service of patients and society. This study aimed to evaluate the perception of professionalism among Latin American physicians in 2024.

**Methodology:**

We conducted an observational, cross-sectional study involving Latin American physicians from various specialties. A telematic survey was administered, which included sociodemographic variables (age, sex, country, specialty) and the modified Kwon et al. professionalism questionnaire, comprising 43 questions across seven dimensions.

**Results:**

Four hundred and twenty-four physicians participated (median age of 40 years, 57.6% female, and 69.8% from clinical specialties) from Paraguay (72.9%), Peru (24.3%), and Cuba (2.8%). A threshold score of 1.223 out of 4 identified the lowest 40% in perceived professionalism. The lowest scoring indicators -reflecting less frequents behavior- were: *I recommend illegal drugs for recreational use* and *I maintain inappropriate body contact during the physical examination*. In contrast, the highest scoring (more frequent) were: *I am late for work* and *I overlook colleagues’ medical errors*. Male physicians demonstrated significantly lower professionalism in three areas: Inappropriate body contact during physical examination, abusive treatment of other healthcare professionals, and discriminatory behavior toward colleagues or patients (adjusted effect).

**Conclusions:**

Sixteen indicators show a significant association with professionalism self-perceived professionalism, with four showing notable differences by sex. Ongoing implementation of such assessment at different professional stages is recommended, along with educational strategies at the university level to reinforce the core values of medical professionalism.

## INTRODUCCIÓN

El concepto de profesionalismo tiene diversas definiciones y, por tanto, es de difícil medición. Incluye criterios como el nivel de habilidad, el buen juicio y el comportamiento cortés que se espera de la persona entrenada para hacer bien su trabajo^1^. Para la Real Academia Española es el *cultivo o utilización de ciertas disciplinas, artes o deportes, como medio de lucro*^2^. En el ámbito médico, es el conjunto de conocimientos, habilidades, principios y valores que sustentan una práctica idónea en el marco de los más elevados estándares de calidad científica, ética y humanística^2^.

El constructo profesionalismo incluye cuatro dimensiones fundamentales: 1) conocimiento especializado, 2) autonomía en la toma de decisiones, 3) compromiso de servicio a la sociedad, y 4) autorregulación^3^^,^^4^. Otros autores incluyen otras dimensiones como el compromiso de respeto a las normas éticas, los ideales y la conciencia profesionales^3^^,^^4^. La autorregulación se refiere al compromiso con el mantenimiento autónomo de conocimientos actualizados o autoformación, con la mejora continua de la competencia profesional. Otra dimensión relacionada al profesionalismo es el altruismo, entendido como la capacidad de equilibrar la disponibilidad de la persona para los demás (pacientes y colegas) con el cuidado de sí misma.

La evaluación de actitudes y valores profesionales se encuentra en los niveles más altos de la pirámide de Miller y requiere métodos sofisticados pues debe medir comportamientos concretos^5^^,^^6^. Existen diversos instrumentos para medir el profesionalismo contiendo diversos dominios y dimensiones^7^^-^^9^.

La medicina puede entenderse como una empresa con una intensa moral interna, donde la ética y el profesionalismo son las bases fundamentales para lograr su objetivo principal, el servicio público. El comportamiento poco profesional puede socavar la confianza organizacional y afectar negativamente la atención a pacientes, el entorno de aprendizaje clínico y el bienestar del profesional de medicina^10^.

Una amenaza para la medicina es la desprofesionalización, que ocurre por la transformación de los sistemas de salud en organizaciones mercantilistas, convirtiendo una noble profesión en un simple oficio corporativo^11^; esto implica que el profesionalismo con *compromiso social* se transforme en un profesionalismo *experto*. Otras amenazas surgen del acelerado avance de la tecnología y sus costes, de los cambios en las fuerzas del mercado, y de los problemas en el acceso y provisión de los servicios de salud, así como de la globalización^3^.

En los últimos años surgió un nuevo concepto: el profesionalismo digital. Este fenómeno se debe a las redes sociales de comunicación por internet, las cuales han afectado la relación cara a cara entre médico y paciente^2^. El uso adecuado de esta nueva tecnología requiere competencia profesional, reputación y responsabilidad, no siempre presentes en los currículos universitarios. El uso inadecuado de internet en la actividad profesional puede llevar, en ocasiones, a generar dilemas morales, sobre todo lo relacionado a la privacidad del paciente, la integridad profesional y el ciberacoso. Los límites entre uso personal de las redes sociales y el profesionalismo digital pueden ser imprecisos^12^^,^^13^.

Dado que el profesionalismo se define como una *manera de actuar* que incluye un conjunto de comportamientos bien codificados, la mayoría de las encuestas solo miden la *intención* o entendimiento (aspectos cognitivos) y no la *acción* (aspectos conductuales) de los principios del profesionalismo^14^^,^^15^. Considerando esta limitación, Hyo-Jin Kwon y col^16^ desarrollaron en Corea un cuestionario dirigido a personal residente de medicina que permite la autoevaluación en los errores que pudieran haber cometido en el ejercicio del profesionalismo^16^. Con ocho dimensiones y 40 preguntas de respuesta múltiple, permite detectar cuánto ha incumplido cada virtud del correcto quehacer del médico, lo que permite identificar los aspectos del profesionalismo que pueden mejorarse a nivel personal e institucional. Este tipo de instrumento brinda información muy útil del estado del ejercicio del quehacer del profesionalismo y, por ende, aplicar las medidas correctivas.

Existen diferencias culturales y temporales en la conceptualización del profesionalismo médico en respuesta a las diversas épocas y al contexto social y cultural de cada país, por lo que los instrumentos para medirlo deben adecuarse a estas situaciones^16^^,^^17^. No existe un instrumento específico para medir el profesionalismo en personal médico latinoamericano, por lo que se utilizará como base el creado por Kwon y col, incluyendo aspectos para evaluar comportamientos del personal médico en relación al uso inapropiado de redes sociales, la discriminación, la comunicación en la era digital, y vestimentas modernas, entre otros.

El objetivo de este estudio es analizar la percepción del profesionalismo en personal médico del Paraguay, Perú y Cuba en 2024 mediante el cuestionario de Kwon y col^16^ modificado.

## MATERIAL Y METODOS

### Diseño y población de estudio

Se aplicó un diseño observacional, descriptivo, de corte transversal, correlacional. La población de estudio estuvo constituida por personas de ambos sexos que cursaran la residencia o fuesen médicos de planta en hospitales del Paraguay, Perú y Cuba entre julio y octubre de 2024. El criterio de inclusión fue la aceptación del consentimiento informado. Fueron excluidos los cuestionarios incompletos. Se utilizó un muestreo no probabilístico, en bola de nieve.

El tamaño de muestra se calculó con el programa estadístico Epi Dat 3.1™. En ausencia de datos previos se estimó un 50% de profesionalismo médico. Para una confianza del 95% y precisión del 5%, el tamaño mínimo a incluir fue 384 cuestionarios.

### Variables e instrumento de medición

Se recogieron las características sociodemográficas: edad (años), sexo (mujer, hombre), país de residencia (Paraguay, Perú, Cuba), especialidad médica (clínica, quirúrgica). Para medir el profesionalismo se utilizaron los indicadores del cuestionario modificado de Kwon y col^16^. El cuestionario contiene 43 preguntas en forma de afirmaciones de aspectos negativos del ejercicio de la profesión que corresponden a siete dimensiones representativas: 1: deshonestidad y práctica insegura en la atención al paciente (9 preguntas), 2: conflictos de interés (4 preguntas), 3: mala conducta en investigación y publicación (9 preguntas), 4: conducta irresponsable e incapacidades del médico (7 preguntas), 5: falta de respeto a profesionales de la salud (4 preguntas), 6: falta de respeto a pacientes y violación de la confidencialidad (4 preguntas) y 7: conductas en la sociedad moderna (6 preguntas). Las respuestas fueron codificadas siguiendo una escala Likert de cuatro puntos: 1 = nunca lo he hecho, 2 = lo hice algunas veces, 3 = lo hago a menudo y 4 = lo hago siempre. El rango de puntuaciones del cuestionario oscila de 43 a 172 puntos, de modo que, a menor puntaje, mayor es el profesionalismo.

Las preguntas del cuestionario fueron inicialmente evaluadas por cinco investigadores expertos para determinar la suficiencia, claridad, coherencia y relevancia de los ítems a medir. El coeficiente de validez de contenido para cada ítem fue del 90%; no se realizaron otros análisis complementarios. Con sus sugerencias se elaboró un cuestionario telemático con el software *Google Forms*™. Los resultados de la prueba piloto con 30 sujetos fueron sometidos a evaluación de la consistencia interna con el programa estadístico *SPSS Statistics 25*™, obteniéndose un alfa de Cronbach de 0,88. Finalmente, el cuestionario se compartió por internet a los investigadores asociados, quienes lo difundieron en los hospitales de cada país participante para acceder a las respuestas del personal médico.

El protocolo del estudio fue aprobado por el Comité de Ética de la Universidad Privada del Este, Paraguay (dictamen 03/2024). Se aseguró el anonimato de las personas participantes. Las personas encuestadas dieron su consentimiento para rellenar el cuestionario y podían retirarse del estudio en cualquier momento sin necesidad de dar explicaciones.

### Análisis estadístico

Las variables fueron sometidas a estadística descriptiva: las cualitativas se expresaron como frecuencias y porcentajes, y las cuantitativas como medidas de tendencia central y de dispersión: media o mediana y desviación estándar (DE) o rango intercuartílico (RIC). Las puntuaciones del cuestionario se dicotomizaron en el percentil 60; se consideró que el 60% de las personas con puntuaciones ≤P_60_ se adhieren a los criterios del profesionalismo y muestran profesionalismo elevado, y que el 40% de las personas con puntuaciones >P_60_ no se adhieren a dichos criterios y muestran profesionalismo bajo.

Los factores sociodemográficos asociados al profesionalismo (variable dependiente) se analizaron mediante análisis bivariado (Chi-cuadrado, *X*^2^), obteniéndose la *odds ratio* (OR) y su intervalo de confianza al 95% (IC 95%). Se realizó un análisis de regresión lineal múltiple para identificar qué preguntas del cuestionario se relacionaban significativamente con el profesionalismo, a partir del coeficiente B y su error estándar (EE). Se utilizó el programa estadístico *SPSS Statistics 25*™. Se consideró significativo todo valor p <0,05.

## RESULTADOS

Se recibieron 424 cuestionarios correctamente cumplimentados por personal médico, con edades comprendidas entre los 17 y 79 años, con predominio de mujeres (57,55%). El Paraguay fue el país que aportó más cuestionarios (73%) y predominaron las especialidades clínicas (70%) (Tabla 1). Las mujeres fueron ligeramente más jóvenes que los hombres (p=0,02), cumplimentaron el 62% de los cuestionarios provenientes del Paraguay y el 44% de los de Perú; y predominaron en ambos tipos de especialidades (Tabla 1).

**Tabla 1:** Características demográficas por sexo de los profesionales médicos encuestados

Características demográficas	Global	Mujeres	Hombres
	n=424	n=244 (57,55%)	n=180 (42,45%)
Edad (años), *mediana (RIC)*	40 (32-54)	39 (31-52)	43 (34-55)
*Procedencia, n (%)*			
Paraguay	309 (72,9)	193 (62,5)	116 (37,5)
Perú	103 (24,3)	5 (43,7)	8 (56,3)
Cuba	12 (2,8)	(50,0)	(50,0)
*Especialidad, n (%)*			
Clínica	296 (69,8)	170 (57,4)	126 (42,6)
Quirúrgica	128 (30,2)	4 (57,8)	4 (42,2)

La Tabla 2 muestra las puntuaciones obtenidas en cada dimensión e ítem del cuestionario de profesionalismo médico. Los indicadores con menor puntuación fueron *Recomiendo drogas ilegales con fines recreativos* y *Mantengo contacto corporal inapropiado durante el examen físico*. Los de mayor puntación fueron *Llego tarde al trabajo* y *Paso por alto los errores médicos de los colegas*. Cuatro indicadores, dos de la dimensión Falta de respeto a los profesionales de la salud (*Ignoro la opinión de otros profesionales de la salud*, y *Trato a otros profesionales de la salud de manera abusiva*), uno de Conducta irresponsable e incapacidades del médico (*Mantengo contacto corporal inapropiado durante el examen físico*), y otro de Conductas en la sociedad moderna (*Ejerzo algún tipo de discriminación con los colegas y pacientes*) presentaron puntuaciones significativamente más elevadas en hombres que en mujeres (Tabla 2).

**Tabla 2:** Respuestas al cuestionario de profesionalismo de Kwon y col^16^

Dimensiones	Puntuación		
Indicadores	Media ± DE		
	Global	Mujer	Hombre
*Deshonestidad y práctica insegura en la atención al paciente*	1,26 ± 0,24	1,25 ± 0,21	1,27 ± 0,28
1. Registro hallazgos físicos no examinados	1,38 ± 0,55	1,37 ± 0,38	1,31 ± 0,61
2. Desatiendo la formación continua de mis conocimientos y destrezas	1,45 ± 0,55	1,13 ± 0,34	1,13 ± 0,36
3. Receto medicamentos en forma innecesaria	1,09 ± 0,30	1,26 ± 0,54	1,27 ± 0,55
4. Indico medicamentos más allá de mi propia capacidad según lo ordenado	1,10 ± 0,32	1,12 ± 0,39	1,16 ± 0,43
5. Proporciono información incorrecta a los pacientes para ocultar la propia ignorancia	1,10 ± 0,30	1,05 ± 0,22	1,07 ± 0,28
6. Evito recetar medicamentos necesarios para obtener beneficio propio	1,08 ± 0,42	1,14 ± 0,48	1,15 ± 0,47
7. No informo la condición médica del paciente al superior para evitar la culpa	1,15 ± 0,48	1,01 ± 0,15	1,03 ± 0,18
8. Oculto el error médico propio	1,38 ± 0,51	1,31 ± 0,48	1,29 ± 0,52
9. Paso por alto los errores médicos de mis colegas	1,66 ± 0,60	1,28 ± 0,46	1,35 ± 0,50
*Conflictos de interés*	1,17 ± 0,26	1,17 ± 0,23	1,18 ± 0,29
10. Recibo dinero o regalos de pacientes	1,14 ± 0,41	1,54 ± 0,56	1,60 ± 0,62
11. Otorgo beneficios o prioridades a los pacientes después de recibir dinero, regalos o favores de ellos	1,08 ± 0,30	1,02 ± 0,16	1,03 ± 0,25
12. Tengo o intento lograr una relación privada con los pacientes	1,19 ± 0,56	1,32 ± 0,55	1,29 ± 0,52
13. Receto ciertas marcas de medicamentos por compensaciones de las empresas farmacéuticas	1,41 ± 0,58	1,86 ± 0,57	1,85 ± 0,64
14. Recomiendo laboratorios o centros diagnósticos por compensaciones económicas	1,07 ± 0,26	1,38 ± 0,50	1,37 ± 0,53
*Mala conducta en investigación y publicación*	1,18 ± 0,22	1,17 ± 0,19	1,18 ± 0,25
15. Hago referencia a artículos no leídos en mis publicaciones	1,27 ± 0,54	1,08 ± 0,28	1,05 ± 0,24
16. Copio y pego párrafos de internet en un artículo sin citar la fuente	1,30 ± 0,49	1,16 ± 0,37	1,15 ± 0,42
17. Utilizo la inteligencia artificial para redactar partes de un artículo	1,35 ± 0,61	1,20 ± 0,59	1,14 ± 0,51
18. Invento datos parciales o completos de una investigación	1,13 ± 0,37	1,16 ± 0,56	1,21 ± 0,32
19. Altero datos y resultados de una investigación	1,07 ± 0,30	2,21 ± 0,42	1,25 ± 0,47
20. Envío el mismo artículo a diferentes revistas después de modificar algunos detalles	1,04 ± 0,24	1,38 ± 0,54	1,36 ± 0,56
21. Compro la autoría de un artículo o solicito la elaboración de una investigación a terceros	1,09 ±0,32	1,40 ± 0,56	1,40 ± 0,61
22. Incluyo como autor a un colega que no contribuyó al trabajo	1,31 ± 0,54	1,10 ± 0,34	1,16 ± 0,41
23. Solicito la propia inclusión como autor a pesar de no haber contribuido en la investigación	1,06 ± 0,25	1,07 ± 0,40	1,09 ± 0,45
*Conducta irresponsable e incapacidades del médico*	1,27 ± 0,21	1,26 ± 0,18	1,29 ± 0,25
24. Llego tarde al trabajo frecuentemente	1,86 ± 0,60	1,33 ± 0,50	1,25 ± 0,48
25. Salgo a escondidas del hospital mientras estaba de servicio o de guardia	1,42 ± 0,53	1,08 ± 0,09	1,02 ± 0,18
26. Salgo del trabajo sin completar las tareas pendientes	1,31 ± 0,47	1,09 ± 0,28	1,05 ± 0,21
27. Dispongo de drogas ilegales para mi uso	1,04 ± 0,28	1,25 ± 0,46	1,23 ± 0,48
28. Recomiendo drogas ilegales con fines recreativos	1,01± 0,13	1,09 ± 0,28	1,10 ± 0,36
29. Mantengo contacto corporal inapropiado durante el examen físico	1,02 ± 0,16	1,26 ± 0,44	1,36 ± 0,50*
*Falta de respeto a los profesionales de la salud*	1,27 ± 0,21	1,25 ± 0,25	1,30 ± 0,30
30. Ignoro la opinión de otros profesionales de la salud	1,49 ± 0,52	1,01 ± 0,12	1,07 ± 0,34*
31. Trato a otros profesionales de la salud de manera abusiva	1,14 ± 0,35	1,07 ± 0,28	1,13 ± 0,36*
32. Critico a colegas médicos u otros profesionales de la salud frente a los pacientes	1,32 ± 0,48	1,06 ± 0,25	1,09 ± 0,36
33. Maltrato verbal o físicamente de médicos o estudiantes más jóvenes	1,16 ± 0,39	1,05 ± 0,25	1,07 ± 0,25
*Falta de respeto a los pacientes y violación de la confidencialidad*	1,25 ± 0,30	1,26 ± 0,29	1,25 ± 0,30
34. Hablo de los pacientes en lugares públicos o fuera del ámbito médico	1,41 ± 0,54	1,40 ± 0,50	1,40 ± 0,59
35. Filtro información médica de los pacientes	1,31 ± 0,50	1,04 ± 0,23	1,09 ± 0,37
36. Aplico un trato descortés con mis pacientes usando palabras o gestos groseros	1,06 ± 0,25	1,11 ± 0,31	1,08 ± 0,29
37. Hablo de los pacientes entre los colegas por diversión o calumniar a los pacientes	1,25 ± 0,47	1,45 ± 0,51	1,45 ± 0,61
*Conductas en la sociedad moderna*	0,98 ± 0,18	0,99 ± 0,17	0,98 ± 0,19
38. Uso las redes sociales para divulgar información confidencial por internet	1,03 ± 0,21	1,38 ± 0,52	1,45 ± 0,54
39. Ejerzo algún tipo de discriminación con los colegas y pacientes	1,07 ± 0,26	1,43 ± 0,50	1,55 ± 0,54*
40. Evito el contacto visual por atender a mi teléfono móvil o pantalla de computadora	1,57 ± 0,58	1,09 ± 0,28	1,05 ± 0,21
41. Utilizo *jeans* rotos (*ripped jeans*) o blusas escotadas en el hospital	1,07 ± 0,27	1,06 ± 0,25	1,11 ± 0,35
42. Acudo al hospital con aspecto desaliñado o poco aseado	1,23 ± 0,44	1,61 ± 0,57	1,72 ± 0,64
43. Poseo múltiples piercings o tatuajes de imágenes agresivas en lugares visibles	1,18 ± 0,56	1,02 ± 0,21	1,06 ± 0,36

DE: desviación estándar; * p < 0,05 (prueba U de Mann-Whitney).

El percentil 60 de la puntuación global fue 1,223 y permitió dividir el profesionalismo médico de las personas encuestadas en alto y bajo. El grupo con alto profesionalismo mostró una edad mediana 3 años superior al de bajo profesionalismo, y estuvo formado más frecuentemente por profesionales de especialidades clínicas y mujeres, aunque ninguna de estas diferencias fue estadísticamente significativa (Tabla 3).

**Tabla 3:** Relación de sexo y tipo de especialidad con nivel de puntuación en profesionalismo médico

Factores	Profesionalismo médico		OR (IC 95%)	p (*X*^2^)
	n (%)			
	Elevado (≥P_60_)	Bajo (<P_60_)		
	n=254	n=170		
Edad (años)*	42 (32-79)	39 (32-69)	-	0,100
*Sexo*				0,500
Femenino (n=244)	149 (61,1)	95 (38,9)	1,1 (0,7-1,6)	
Masculino (n=180)	105 (58,3)	75 (41,7)		
*Especialidad*				0,091
Clínica (n=296)	185 (62,5)	111 (37,5)	1,4 (0,9-2,1)	
Quirúrgica (n=128)	69 (53,9)	59 (46,1)		

*: mediana (rango intercuartílico) comparado mediante U de Mann-Whitney.

Mediante el análisis de regresión lineal se identificaron 17 preguntas del cuestionario (39%) estadísticamente relacionadas con la percepción del profesionalismo en médicos (Tabla 4). Cinco correspondieron a la dimensión *Deshonestidad y práctica insegura en la atención al paciente*; las dimensiones *Conflictos de interés* y *Mala conducta en investigación y publicación* aportaron tres cada una, *Falta de respeto a los profesionales de la salud* y *Conductas en la sociedad moderna* aportaron dos, y *Conducta irresponsable e incapacidades del médico* y *Falta de respeto a los pacientes y violación de la confidencialidad* aportaron una.

**Table:** 

Preguntas	B (EE)	p
1. Registro hallazgos físicos no examinados	0,045 (0,024)	0,059
2. Desatiendo la formación continua de mis conocimientos y destrezas	0,098 (0,045)	0,03
3. Receto medicamentos en forma innecesaria	0,043 (0,029)	0,134
4. Indico medicamentos más allá de mi propia capacidad según lo ordenado	0,135 (0,04)	0,001
5. Proporciono información incorrecta a los pacientes para ocultar la propia ignorancia	-0,04 (0,065)	0,54
6. Evito recetar medicamentos necesarios para obtener beneficio propio	0,048 (0,032)	0,14
7. No informo la condición médica del paciente al superior para evitar la culpa	0,195 (0,093)	0,037
8. Oculto el error médico propio	0,231 (0,031)	<0,001
9. Paso por alto los errores médicos de los colegas	0,087 (0,032)	0,007
10. Recibo dinero o regalos de los pacientes	-0,003 (0,026)	0,898
11. Otorgo beneficios o prioridades a los pacientes después de recibir dinero, regalos o favores de ellos	-0,335 (0,096)	<0,001
12. Tengo o intento lograr una relación privada con los pacientes	0,053 (0,03)	0,076
13. Receto ciertas marcas de medicamentos por compensaciones de las empresas farmacéuticas	0,055 (0,027)	0,042
14. Recomiendo laboratorios o centros diagnósticos por compensaciones económicas	0,126 (0,037)	0,001
15. Hago referencia a artículos no leídos en mis publicaciones	0,08 (0,057)	0,159
16. Copio y pego párrafos de internet en un artículo sin citar la fuente	0,115 (0,044)	0,01
17. Utilizo la inteligencia artificial para redactar partes de un artículo	-0,002 (0,027)	0,939
18. Invento datos parciales o completos de una investigación	0,052 (0,026)	0,047
19. Altero datos y resultados de una investigación	-0,012 (0,036)	0,742
20. Envío el mismo artículo a diferentes revistas después de modificar algunos detalles	0,053 (0,033)	0,105
21. Compro la autoría de un artículo o solicito la elaboración de una investigación a terceros	0,077 (0,03)	0,01
22. Incluyo como autor a un colega que no contribuyó al trabajo	0,029 (0,05)	0,56
23. Solicito mi propia inclusión como autor a pesar de no haber contribuido en la investigación	0,06 (0,034)	0,083
24. Llego tarde al trabajo frecuentemente	-0,037 (0,034)	0,268
25. Salgo a escondidas del hospital mientras estaba de servicio o de guardia	-0,189 (0,143)	0,187
26. Salgo del trabajo sin completar las tareas pendientes	0,08 (0,062)	0,202
27. Dispongo de drogas ilegales para mi uso	0,102 (0,039)	0,01
28. Recomiendo drogas ilegales con fines recreativos	0,042 (0,049)	0,392
29. Mantengo contacto corporal inapropiado durante el examen físico	0,037 (0,033)	0,258
30. Ignoro la opinión de otros profesionales de la salud	-0,178 (0,076)	0,02
31. Trato a otros profesionales de la salud de manera abusiva	0,058 (0,053)	0,275
32. Critico a colegas médicos u otros profesionales de la salud frente a los pacientes	-0,008 (0,057)	0,89
33. Maltrato verbal o físicamente a médicos o estudiantes más jóvenes	0,27 (0,063)	<0,001
34. Hablo de los pacientes en lugares públicos o fuera del ámbito médico	0,118 (0,031)	<0,001
35. Filtro información médica de los pacientes	-0,026 (0,065)	0,691
36. Aplico un trato descortés con mis pacientes usando palabras o gestos groseros	-0,02 (0,053)	0,705
37. Hablo de los pacientes entre los colegas por diversión o calumniar a los pacientes	-0,02 (0,03)	0,496
38. Uso las redes sociales para divulgar información confidencial por internet	0,059 (0,032)	0,068
39. Ejerzo algún tipo de discriminación con los colegas y pacientes	0,071 (0,03)	0,02
40. Evito el contacto visual por atender a mi teléfono móvil o pantalla de computadora	0,08 (0,056)	0,149
41. Utilizo *jeans* rotos (*ripped jeans*) o blusas escotadas en el hospital	0,13 (0,055)	0,019
42. Acudo al hospital con aspecto desaliñado o poco aseado	0,033 (0,026)	0,217
43. Poseo múltiples piercings o tatuajes de imágenes agresivas en lugares visibles	-0,095 (0,056)	0,092

B: coeficiente; EE: error estándar.

Mediante el análisis de regresión lineal aplicado por sexo a los indicadores estadísticamente significativos en el análisis bivariado, solo tres preguntas permanecieron significativas (Tabla 5): *Mantengo contacto corporal inapropiado durante el examen físico*, *Trato a otros profesionales de la salud de manera abusiva* y *Ejerzo algún tipo de discriminación con los colegas y pacientes.* Todas ellas tienen signo negativo, correspondiendo a menor profesionalismo en los varones.

**Tabla 5:** Preguntas del cuestionario más relacionadas con el profesionalismo médico (regresión lineal múltiple)

Preguntas	B (EE)	p
29. Mantengo contacto corporal inapropiado durante el examen físico	-0,247 (0,037)	<0,001
30. Ignoro la opinión de otros profesionales de la salud	0,008 (0,066)	0,902
31. Trato a otros profesionales de la salud de manera abusiva	-0,296 (0,056)	<0,001
39. Ejerzo algún tipo de discriminación con los colegas y pacientes	-0,276 (0,034)	<0,001

EE: error estándar.

## DISCUSIÓN

Los indicadores del cuestionario de Kwon HJ *et al* modificado halló 254 médicos con elevada percepción de adecuación a las actitudes profesionales. En comparación con otros estudios, en Corea se halló que 50% los médicos de guardia se adhieren a los preceptos del profesionalismo^18^. En Arabia, 63% de los médicos incumplen normas de profesionalismo al menos una vez al mes^10^. Una encuesta de 102 establecimientos de salud de Estados Unidos informó que 77% de los profesionales de la salud habían sido testigos del comportamiento poco profesional de los médicos y 65% lo presenciaron al menos cinco o seis ocasiones por año^10^.

Pero más allá que cuantificar el nivel de este constructo, lo importante es identificar las dimensiones e indicadores que muestran más bajo cumplimiento. Los cuestionarios pueden evaluar el conocimiento de los principios del profesionalismo, el razonamiento moral y la toma de decisiones, pero no puede evaluar lo que podría hacer en la práctica real^19^. La evaluación del profesionalismo es difícil de realizar. Es un concepto multidimensional, por lo que para su determinación requiere una combinación de herramientas. No se cuenta con un único instrumento de medición que tenga validez, fiabilidad y funcionalidad^3^^,^^4^.

Los resultados de la evaluación del profesionalismo pueden verse afectados por la edad, el sexo, la formación académica, el estado civil, el grado académico y la especialidad^4^^,^^16^. Estudios previos realizados en Paraguay han hallado que los médicos residentes varones ejercen más maltratos a los subalternos que sus congéneres femeninos^20^. Entre estos se hallaron las insinuaciones verbales sexuales o comentarios obscenos, mostrar un lenguaje corporal o gestos ofensivos de tipo sexual y la discriminación por género. Estas diferencias por sexo deberían investigarse con estudios cualitativos, aunque se asume que se deben a la cultura machista que predomina en la población latinoamericana.

Se ha descrito que residentes de ginecología y obstetricia obtienen más altos puntajes al evaluar el altruismo, el honor y la integridad en comparación con residentes de cirugía^9^; en nuestro estudio, el personal de áreas quirúrgicas tendió a mostrar menor respeto a las actitudes profesionales. Una investigación realizada en Paraguay demostró que residentes de cirugía y especialidades afines sufren maltrato más frecuentemente que sus homólogos de especialidades clínicas^20^. También destacó la costumbre de llegar tarde al trabajo y salir a escondidas del hospital. El tutor o mentor es el responsable directo de fomentar estas conductas responsables, siempre con su ejemplo^21^. Estos comportamientos deben tener una explicación y ameritan una evaluación cualitativa, ya que con seguridad afectan al funcionamiento de los servicios médicos.

En este estudio, el contacto corporal inapropiado durante el examen físico y la intención lograr una relación privada con los pacientes fueron indicadores de baja percepción de profesionalismo. En comparación con otras profesiones, los profesionales de medicina pueden lograr contactos cercanos con otras personas más frecuentemente, si bien los códigos de ética y moral tradicionales son claros al respecto y se consideran conductas inapropiadas para cualquier sociedad y tiempo^22^. El profesionalismo y sus dimensiones se diseñaron con la homogeneidad histórica de la medicina académica^23^. Actualmente se considera falta de profesionalismo a la discriminación por diversos motivos como las ideologías culturales, religiosas o políticas, la vestimenta, el estilo del corte de pelo, y la manera de hablar o actuar, entre otras^24^^,^^25^.

Sin embargo, existen costumbres novedosas para los tiempos actuales, tales como asistir a los hospitales sin batas blancas, desaseados, con múltiples tatuajes o *piercing*, blusas escotadas o *jeans* rotos. Estos indicadores fueron incluidos en la encuesta y mostraron un uso frecuente, sobre todo en médicos jóvenes. Estos rasgos son aspectos que no son mal vistos ni por galenos ni por pacientes jóvenes, lo que indica que algunos principios del profesionalismo tienden a evolucionar con el tiempo^2^^,^^23^.

Otra dimensión muy actual se refiere al área de las publicaciones científicas. Publicar artículos científicos constituye un determinante para ascender en la carrera profesional y obtener reconocimiento por sus pares, por lo que existe presión por publicar. En este estudio la percepción respecto de las normas éticas de la publicación indicó uso inapropiado de las fuentes de información, aplicación de inteligencia artificial para la redacción, y autoría por cortesía, entre otros aspectos, probablemente en relación a que el personal médico se ve forzado a publicar. Pero la creación intelectual requiere honestidad, transparencia y responsabilidad de los autores^26^.

En el campo médico, internet y las redes sociales se utilizan frecuentemente para compartir información entre colegas, para educación continua y para campañas de promoción de la salud y prevención de enfermedades^13^. En los últimos años ha aumentado de forma importante el uso de redes sociales por parte de profesionales sanitarios sin adherirse a los principios del profesionalismo, llevando incluso a infracciones por mala conducta profesional. Esto puede deberse a que no existen normas del profesionalismo en el contexto digital, concepto conocido como e-profesionalismo^13^. Desafortunadamente, aún existen muchas inconsistencias en los significados conceptuales y límites de esta dimensión para garantizar que los profesionales de la salud mantengan altos estándares mientras practican en el ámbito digital. Este desafío obliga a seguir investigando este tema en los próximos años.

En Corea, más de la mitad de los médicos encuestados informaron haber experimentado una conducta poco profesional a pesar de sus connotaciones negativas. Predominaron las de *manipular los datos de la investigación para obtener los resultados deseados*, *prescribirse medicamentos, incluso antipsicóticos, a uno mismo*, *mantener relaciones sociales con el paciente*, *tocar una parte del cuerpo que no es necesaria para el diagnóstico al examinar a un paciente* y *fotografiar o grabar un vídeo de una lesión para publicarlo en una red social sin el consentimiento del paciente*^18^. En Irán se detectó una elevada proporción de conductas poco profesionales en residentes de medicina^27^, identificándose que la participación en las mismas se debe a la debilidad del reconocimiento de la responsabilidad personal. Entre los factores sistémicos se identificó la deficiencia de un currículo explícito, además de la ausencia de mecanismos de denuncia de conductas poco profesionales^27^. Este aspecto no se evaluó en esta investigación, quedando pendiente para próximos estudios. Algunos autores sugieren implementar un libro de registro de incidentes de falta de profesionalismo y los informes de incidentes críticos^3^^,^^5^.

El profesionalismo es un conjunto de actitudes, valores, comportamientos e interacciones que actúan como base del contrato del profesional de la salud con la sociedad^13^. Es importante monitorear los comportamientos no profesionales o disruptivos pues existen estrategias para compensar o mejorar las actitudes negativas^16^^,^^9^. Mejorar el profesionalismo en la profesión médica requiere conocer la frecuencia, los tipos, las fuentes y los objetivos del comportamiento no profesional para refinar los programas y estrategias organizacionales para abordarlos y prevenirlos^6^^,^^10^^,^^28^.

El ejercicio actual de la medicina se enfrenta a múltiples desafíos relacionados con los cambios de paradigmas, el uso creciente de la tecnología, o las exigencias de las corporaciones públicas y privadas^2^^,^^13^. El personal médico residente no se encuentra ajeno a estos dilemas pues esta etapa de la formación de postgrado es un período crítico en el desarrollo de la identidad profesional y adquisición de conocimientos, habilidades y conductas, por lo que representa un desafío para los docentes operativizar el aprendizaje de los comportamientos profesionales^6^^,^^16^. Los residentes deben vivir y conciliar su entorno con lo que debería ser el mundo de un profesional de la medicina^11^. Es obligación de las instituciones hospitalarias y educativas crear un ambiente de trabajo adecuado y fomentar en el personal médico las virtudes humanas, a sabiendas de que es un tema desafiante y complejo^7^^,^^28^.

Las limitaciones de esta investigación son varias. Es sabido que las personas encuestadas tienden a dar respuestas ideales o agradables para la sociedad, aunque sean anónima^7^^,^^15^. A consecuencia del diseño observacional y transversal aplicado, no se pueden obtener inferencias. Además, el muestreo no fue aleatorio. No obstante, como fortaleza, es un estudio multinacional y con importante tamaño de muestra. Además, utilizó un cuestionario que fue previamente validado por otros investigadores. Otra limitación es el punto de corte utilizado para clasificar a los encuestados en dos niveles de profesionalismo. El mismo se estableció considerando la mediana y el rango intercuartílico ± 10, como fue aplicado por otros autores^29^. Si bien esta decisión puede condicionar los resultados presentados, es habitual estimar que los sujetos que sobrepasen el percentil 60 o 70 se encuentran fuera de rango de lo esperado en esa muestra.

En conclusión, el promedio de profesionalismo de la muestra fue 1,20 (DE=0,17), teniendo en cuenta que 1 es el nivel más elevado de este constructo. Se halló una diferencia significativa por sexo en algunos indicadores, donde los varones más frecuentemente mantuvieron un contacto corporal inapropiado durante el examen físico, trataron a otros profesionales de la salud de manera abusiva y ejercieron algún tipo de discriminación con los colegas y pacientes.

Se recomienda seguir aplicando este tipo de encuestas para observar la evolución de los rasgos medidos, agregar más indicadores considerando los usos y costumbres de la tecnología y la vida moderna y, a nivel universitario, insistir en generar conciencia sobre los valores del profesionalismo.

## Data Availability

Se encuentran disponibles bajo petición al autor de correspondencia.
